# The Therapeutics of the Thymus Gland

**Published:** 1900-08

**Authors:** 


### The Therapeutics of the Thymus Gland.

Dr. R. S. Solis-Cohen, of Philadelphia, spoke on this topic. He said that it had long been known that in cases of co-called thymic asthma children died through some mysterious influence exerted by the thymus gland, and while many authors had written learnedly on the subject, he personally felt that they had shed very little light on it. One reason for contradictory statements from different competent observers was to be found in the varying qualities of different preparations. He had addressed a letter of inquiry to the principal manufacturers of these products, and from their replies he had learned that no two of the American products were alike. Mr. David Owen's supposed cure of a case of exophthalmic goitre by the administration of thyroid extract turned out, on further investigation, to be a case in which, without his knowledge, the butcher had substituted thymus gland. This had naturally led to the deliberate use of thymus gland from the calf. In one of his cases that showed no improvement, it was discovered that the thymus gland from the sheep had been used instead. In a case of severe exophthalmic goitre, reported by Mr. Todd, of England, recovery ensued after the use of the thymus gland from the calf, although previously it had proved most obstinate under all the approved methods of medication. Dr. Cohen said that he had personally seen many good results from the use of this gland, but he had recently abandoned it in favor of suprarenal gland extract, which acted still better. Some cases presented symptoms which were best controlled by the suprarenal extract; others yielded better to the thymus extract. On the whole, a combination of the two had seemed to work better than either one alone. Physiologists had not given us much light on this matter. We were told that the active principle of the thymus gland when injected into the veins caused extensive vascular clotting, and hence this remedy has been used as a styptic, and had been recommended in cases of hæmophilia. This extract had also been used and recommended in leukæmia, rickets, and rheumatoid arthritis. Kinnicutt and others had claimed good results from it in pulmonary tuberculosis. Chittenden has shown that the thymus gland contained an exceedingly large proportion of phosphorus, which might possibly explain some of the good results claimed for it.—*N. Y. Medical Record.*

